# A Brazilian university hospital position regarding transplantation criteria for HIV-positive patients according to the current literature

**DOI:** 10.6061/clinics/2019/e941

**Published:** 2019-03-25

**Authors:** Lígia Camera Pierrotti, Nadia Litvinov, Silvia Figueiredo Costa, Luiz Sérgio Fonseca de Azevedo, Tânia Mara Varejão Strabelli, Silvia Vidal Campos, Fatuma Catherine Atieno Odongo, Jose Otto Reusing-Junior, Alice Tung Wan Song, Max Igor Banks Ferreira Lopes, Marjorie Vieira Batista, Marta Heloisa Lopes, Natalya Zaidan Maluf, Hélio Helh Caiaffa-Filho, Maura Salarolli de Oliveira, Heloisa Helena de Sousa Marques, Edson Abdala

**Affiliations:** I Divisao de Molestias Infecciosas, Hospital das Clinicas HCFMUSP, Faculdade de Medicina, Universidade de Sao Paulo, Sao Paulo, SP, BR; II Subcomite de Infeccao em Imunodeprimidos, Hospital das Clinicas HCFMUSP, Faculdade de Medicina, Universidade de Sao Paulo, Sao Paulo, SP, BR; III Instituto da Crianca (ICr), Hospital das Clinicas HCFMUSP, Faculdade de Medicina, Universidade de Sao Paulo, Sao Paulo, SP, BR; IV Departamento de Molestias Infecciosas, Faculdade de Medicina FMUSP, Universidade de Sao Paulo, Sao Paulo, SP, BR; V Servico de Transplante Renal, Hospital das Clinicas HCFMUSP, Faculdade de Medicina, Universidade de Sao Paulo, Sao Paulo, SP, BR; VI Nucleo de Transplante Cardiaco, Instituto do Coracao (InCor), Hospital das Clinicas HCFMUSP, Faculdade de Medicina, Universidade de Sao Paulo, Sao Paulo, SP, BR; VII Servico de Pneumologia, Grupo de Transplante Pulmonar, Instituto do Coracao (InCor), Hospital das Clinicas HCFMUSP, Faculdade de Medicina, Universidade de Sao Paulo, Sao Paulo, SP, BR; VIII Divisao de Transplante de Figado e Orgaos do Aparelho Digestivo, Departamento de Gastroenterologia, Hospital das Clinicas HCFMUSP, Faculdade de Medicina, Universidade de Sao Paulo, Sao Paulo, SP, BR; IX Servico de Imunologia, Hospital das Clinicas HCFMUSP, Faculdade de Medicina, Universidade de Sao Paulo, Sao Paulo, SP, BR; X Servico de Biologia Molecular, Hospital das Clinicas HCFMUSP, Faculdade de Medicina, Universidade de Sao Paulo, Sao Paulo, SP, BR; XI Grupo Controle de Infeccao, Hospital das Clinicas HCFMUSP, Faculdade de Medicina, Universidade de Sao Paulo, Sao Paulo, SP, BR

**Keywords:** HIV, TRANSPL, Immunossupression

## Abstract

Human immunodeficiency virus (HIV) infection was considered a contraindication for solid organ transplantation (SOT) in the past. However, HIV management has improved since highly active antiretroviral therapy (HAART) became available in 1996, and the long-term survival of patients living with HIV has led many transplant programs to reevaluate their policies regarding the exclusion of patients with HIV infection.

Based on the available data in the medical literature and the cumulative experience of transplantation in HIV-positive patients at our hospital, the aim of the present article is to outline the criteria for transplantation in HIV-positive patients as recommended by the Immunocompromised Host Committee of the Hospital das Clínicas of the University of São Paulo.

## INTRODUCTION

Human immunodeficiency virus (HIV) infection was considered a contraindication for solid organ transplantation (SOT) in the past. Initial studies of patients who underwent SOT reported worse outcomes in HIV-positive individuals compared to HIV-negative individuals. A particular concern was that posttransplant drug-induced immunosuppression could enhance the immunosuppressive state already induced by HIV thus increasing morbidity, mortality and organ waste 1.

The prognosis of HIV infection has improved dramatically since highly active antiretroviral therapy (HAART) became available in 1996. The impact of HAART has been supported by the significant decrease in morbidity and mortality, with a 10-year survival rate exceeding 90% 2. Consequently, the long-term survival of HIV-infected patients as well as chronic comorbidities other than opportunistic infection have increased. Some of these diseases, such as chronic kidney disease, coronary artery disease, diabetes mellitus and liver failure, have become important contributors of the poor prognosis for these patients. Despite adequate control of viral replication with antiretroviral drugs 3,4, these comorbidities can lead to end-stage organ failure with no alternative medical treatment except transplantation.

Improvements in HIV management and the long-term prognosis of patients living with HIV have led many transplant programs to reevaluate their policies regarding the exclusion of patients with HIV infection. The cumulative evidence of transplantation in HIV-positive patients in the era of HAART has shown that immunosuppressive therapy did not negatively impact the posttransplant course of HIV infection 5.

Most of the reported HIV-infected patients who underwent SOT had to fulfill strict inclusion criteria relating to a stable immunological and virological status. These patients did not experience HIV replication after transplantation but presented a transient decrease in CD4+ T-lymphocyte counts during the first months posttransplant, but these counts rapidly returned to baseline values. The other inclusion criteria not related to HIV status were identical in HIV-positive and HIV-negative patients 6.

Increased knowledge on appropriate HAART regimens for SOT recipients has also contributed to the reported improved outcomes. Integrase strand transfer- and nucleoside reverse transcriptase inhibitor-based antiretroviral therapies have been associated with better outcomes than protease inhibitor-based therapies due to fewer drug interactions 6,7. These two classes of antiretroviral drugs are not substrates of CYP450; therefore, they are preferred and can be safely used during the posttransplant period. If the pretransplantation antiretroviral regimen is changed, it is important to wait at least 6 months before transplantation.

Reported experiences of transplantation in the setting of HIV infection are based mostly on kidney and liver transplantation, with much less reported experience with the transplantation of other solid organs. Nevertheless, significant challenges have been raised when considering HIV-positive candidates for SOT, including the impact of coinfection of HIV with hepatitis C virus (HCV), hepatitis B virus (HBV) or other viruses on the risk of opportunistic infection and malignancy rates after transplantation, as well as the direct effect of HIV itself and the potential of drug toxicities and interactions.

Based on the available data in the medical literature and the cumulative experience of transplantation in HIV-positive patients at our hospital, the aim of the present article is to outline the criteria for transplantation in HIV-positive patients as recommended by the Immunocompromised Host Committee of the Hospital das Clínicas of the University of São Paulo.

### Kidney Transplant

HIV-positive patients are at increased risk for end-stage renal disease (ESRD), and kidney transplantation has been an effective and safe treatment in this scenario. Renal disease in this population is related to multiple causes, including HIV-associated nephropathy (HIVAN), acute renal injury, renal toxicity of antiretroviral drugs and comorbid conditions such as HCV, hypertension and diabetes 8. HIV infection and diabetes increase the risk of ESRD, particularly among black individuals. In populations without HIV or diabetes, Choi et al. 9 reported a 4- to 5-fold increase in the risk of ESRD in black individuals compared to white individuals.

Several publications from the pre-HAART era corroborated the poor results among HIV-infected individuals who underwent kidney transplantation, with a higher mortality related to acquired immune deficiency syndrome (AIDS) during the posttransplant period (ranging from 33% to 80%) compared to the overall mortality among those maintained in renal replacement therapy 1.

Experiences regarding kidney transplantation in HIV-infected individuals in the era of HAART have changed dramatically. Recent studies reported that the appropriate selection of HIV-positive candidates for kidney transplant has contributed to similar graft and patient survival rates in HIV-positive and HIV-negative patients.

To date, the largest published study prospectively evaluated 150 HIV-infected individuals who underwent kidney transplantation and were followed for up to five years at 19 transplant centers in the United States. The participants were carefully selected using criteria of a CD4+ T-cell count at least 200 cells/mm^3^ and an undetectable HIV viral load. The one- and three-year patient survival rates were 94.6% and 88.2%, respectively, and the graft survival rates were 90.4% and 73.7%, respectively. The graft survival rates were similar to those reported for kidney transplant recipients aged ≥65 years in the Scientific Registry of Transplantation Recipients (SRTR) database. However, there were higher rates of acute rejection at 1 and 3 years posttransplantation (31% and 41%, respectively) 10.

A number of additional studies have demonstrated that HIV-positive patients have patient and graft survival rates similar to those of HIV-negative patients undergoing renal transplantation in the HAART era 11-16, while some authors have also reported elevated acute rejection rates ranging from 15% 17 to 44% 18.

A large retrospective review of the United Network for Organ Sharing (UNOS) database from 2004 to 2006 comparing 100 HIV-positive and 36,492 HIV-negative kidney transplant recipients showed no differences in patient survival rates (95.4% *vs* 96.2% *p*=0.32). However, the death-censored graft survival rate was significantly lower in HIV-positive patients (87.9% *vs* 94.9%, *p*=0.03). The donor's age and history of hypertension, a cold ischemia time of at least 16 hours and delayed graft function were associated with a greater than fourfold increase in allograft loss among HIV-positive patients 19. These findings suggest that marginal kidney allografts are more prone to nephrotoxicity, which is a particularly greater threat in HIV-positive individuals.

Two recent studies have evaluated the effect of HIV-HCV coinfection on the outcomes of kidney transplant recipients. The authors found that HIV-HCV coinfected patients had inferior graft and patient survival rates compared to those of HIV- or HCV-monoinfected individuals 20,21. In one of these studies, the authors reported 10-year outcomes of kidney transplant recipients from 2002 to 2012 using the SRTR database. Monoinfected HIV-positive recipients had similar 5- and 10-year graft survival rates when compared to matched HIV- and HCV-negative controls. In addition, compared to HIV-HCV coinfected patients, both HIV- and HCV-monoinfected matched controls presented higher patient survival rates at 5 and 10 years after transplantation. HCV coinfection, panel reactive antibodies >80%, acute rejection episodes and cold ischemia time >10 hours were independent risk factors for graft loss 20.

The largest Brazilian report of kidney transplantation in 53 HIV-infected recipients and 106 HIV-negative controls was published by Vicari et al. 22. In this multicenter study, the HIV-positive group presented a higher incidence of delayed graft function (*p*=0.044), acute rejection (*p*=0.036) and median number of infections per patient (*p*=0.018) compared to the control group. The HIV-infected patient group presented lower rates of 1-year patient survival (90.6% *vs* 100%, *p*<0.001) and graft survival (90.4% *vs* 98.1%, *p*=0.004) than the non-HIV-infected control group, but those numbers were considered acceptable by the authors. In all centers, the HIV-positive patients accepted for transplantation were clinically stable on HAART with CD4+ cell counts above 200 cells/mm^3^ and undetectable HIV viral loads.

There is limited reported experience with simultaneous kidney-pancreas transplantation in HIV patients 23-25, and, only recently, the first case of pancreas transplant alone was reported 26. However, the evidence has been sufficient to support the feasibility of pancreas transplantation (either alone or in association with kidney) for people living with HIV.

### Liver Transplant

Liver disease is currently an important cause of non-AIDS-related death in HIV-positive individuals 3 and is associated with several factors, including hepatitis virus coinfection, antiretroviral-related liver toxicity, alcohol abuse, nonalcoholic cirrhosis, and hepatocellular carcinoma (HCC). Chronic HCV and HBV coinfection is common in HIV-infected patients, with estimated HIV-HCV coinfection rates of approximately 33%, but rates can reach up to 70% in patients progressing to cirrhosis 27,28.

Several studies have shown that HIV-infected patients present higher mortality rates after their first liver disease decompensation, and the absolute value of the Model For End-Stage Liver Disease (MELD) score was associated with significantly poorer survival rates in comparison with the scores of HIV-negative patients 29-31. Murillas et al. 32 showed that the mortality rate for a given MELD score in HIV-infected patients was similar to the mortality rate in non-HIV-infected patients with a MELD score that was approximately 10 points higher. In addition, some studies suggest that HIV-infected patients present a more aggressive course of HCC than non-HIV-infected patients 33,34. This scenario has contributed to the fact that few HIV-positive patients are accepted for liver transplantation. Moreover, those who are accepted have a reduced probability of successfully undergoing transplantation due to poor clinical conditions 27.

Initial studies performed before the advent of HAART showed poor results 35,36. A French study demonstrated a 7-year survival rate of 36% among 11 HIV-infected liver transplant recipients compared to almost 70% among non-HIV-infected liver transplant recipients during the same period 37. Even in the post-HAART era, a cohort study with the UNOS database evaluating the outcomes of liver transplantation in HIV-positive individuals who underwent transplantation between 1997 and 2006 showed inferior overall survival rates among HIV-positive patients (n=138) compared to those of HIV-negative patients (n=30520). Survival rates at 2 and 3 years posttransplantion were 70% and 60% in HIV-positive recipients compared to 81% and 77%, respectively, in the controls (*p*<0.05) 38.

Liver transplantation outcomes in HIV-HCV coinfection patients have been associated with higher mortality rates. HCV recurrence represented the major challenge in HIV-HCV coinfected patients in the past 15. Two important prospective studies, one in Spain 39 and the other in the United States 40, performed with HIV-HCV coinfected patients undergoing liver transplantation showed similar results. HIV-HCV coinfected patients had lower patient and graft survival rates and more acute cellular rejection than did monoinfected HCV recipients in both studies. Nevertheless, the use of the newer directly acting antiviral (DAA) agents for HCV treatment, resulting in a sustained viral response (SVR), has changed this scenario dramatically. Data have shown that the use of new DAA agents in HIV-HCV coinfected patients following liver transplantation is associated with improved SVR rates 41,42. Moreover, both pretransplant and early posttransplant treatment have changed this scenario 43,44.

In contrast, patients with liver disease associated with chronic HBV and nonviral etiologies undergoing liver transplantation have presented excellent results with a reported overall survival rate of 100% at 5 years 28. Although the reported experience in liver transplantation for HCC in HIV-positive patients is limited, the comparison of the results of liver transplantation for HCC has shown similar results between HIV-positive and HIV-negative recipients regarding HCC recurrence, disease-free survival time and overall survival rates 45,46.

### Heart Transplant

Cardiovascular disease is a significant cause of morbidity in patients living with HIV, and heart transplantation has become an acceptable therapeutic modality in selected HIV-positive patients. The 2016 International Society for Transplantation listing criteria for heart transplantation has considered including HIV-positive candidates in special circumstances. Centers performing heart transplantation in this population should have structured protocols with multidisciplinary teams, adequate access to pharmacologic expertise, therapeutic drug monitoring for immunosuppressants, and laboratory access to antiviral drug resistance testing as needed 47.

However, the current experience is limited to a few reported cases and small series. Some of these reported cases occurred in patients who underwent heart transplantation at the time when antibody-based HIV testing was not routinely available 48,49. In the pre-HAART era, Castel et al. 50 published a case report of a 39-year-old HIV-positive male patient in Spain with a high CD4 count and undetectable HIV viral load receiving an antiretroviral regimen based on three analog reverse transcriptase inhibitors (zidovudine, lamivudine and abacavir) and who underwent heart transplantation. The patient had one episode of rejection without infectious episodes and was alive 3 years posttransplant.

Additional case reports in the post-HAART era have been documented; the majority of these patients underwent heart transplantation with a high CD4+ T-cell count and undetectable HIV viral load and presented satisfactory outcomes without AIDS-related complications after transplantation 51.

In one case report, a 39-year-old patient was diagnosed with HIV infection nine years before heart transplantation. He had a history of opportunistic diseases, including *Pneumocystis jirovecii* pneumonia at the time of HIV diagnosis and disseminated Kaposi sarcoma diagnosed a few months afterwards. In the posttransplantation follow-up period of 30 months, this patient experienced occasional decreases in the CD4+ T-cell count to less than 100 cells/mm^3^, but he did not present any opportunistic disease. The main complications after transplantation were recurrent episodes of rejection 52.

The largest case series was reported by the Columbia Presbyterian Medical Center with 5 HIV-positive patients who underwent heart transplantation. All patients had a CD4+ T-cell count above 400 cells/mm^3^ and an undetectable HIV viral load; all patients were alive with a mean follow-up of 30 months, none of whom presented significant AIDS-related infections or complications after the transplant procedures 53.

### Lung Transplant

The first case of a successful lung transplant in an HIV-infected patient with cystic fibrosis was described in 2009 54. More recently, three additional cases of lung transplant in HIV-infected patients were reported. Although one patient developed recurrent and refractory acute rejection leading to bronchiolitis obliterans syndrome, the other two only experienced mild acute rejection with good quality of life and good lung function after transplantation 55.

At the end of 2014, the International Society of Heart and Lung Transplantation considered the possibility of including HIV-positive individuals on the waiting list for lung transplantation 56. The document, however, recommends that the lung transplantation procedure in HIV-positive patients should be limited to centers with expertise in the care of these patients.

Reported experiences are still scarce and based on very few cases. At the same time, the majority of lung transplant centers consider listing HIV-positive lung transplant candidates on a case-by-case basis.

### Hematopoietic Stem Cell Transplantation

Despite improvement in the control of HIV infection after the introduction of HAART, HIV-infected patients have a considerably higher risk (15–24 times greater) of developing hematological malignancies , including Hodgkin (HL) and non-Hodgkin lymphoma (NHL), acute leukemia and myelodysplastic syndromes, than non-HIV-infected patients 57. Studies have shown similar prognoses among patients with HIV-related and non-HIV-related lymphomas 57,58.

Hematopoietic stem cell transplantation (HSCT) has been suggested as a treatment option for HIV-infected patients with hematological diseases, but before the era of HARRT, these procedures were mostly unsuccessful. After the introduction of HAART, several transplantation centers have published their experience with autologous HSCT in patients with HIV-related lymphoma (HRL) with some success. A retrospective study of the European Group for Blood and Marrow Transplantation Lymphoma Working Party found no difference in the outcome of HIV-infected and noninfected patients with HL and NHL who underwent autologous HSCT 59.

A case-control study including HIV-positive and HIV-negative patients with NHL observed similar long-term outcomes between cases and controls. The nonrelapse mortality and the 2-year disease-free survival were similar between the HIV-positive group and HIV-negative group (11% *versus* 4%, *p*=0.18, and 75% *versus* 56%, *p*=0.33, respectively) 60.

A recent clinical trial involving 40 transplants in patients with HRL and 151 controls showed that the outcome differences between HIV-infected patients and controls were not statistically significant. Among HRL patients, the 1-year and 2-year overall survival rates were 87% and 82%, respectively. In this trial, 20% of the patients had a detectable viral load at the time of transplantation, and the mean pretransplant CD4+ T-cell count was 249 cells/mm^3^ (range 39–797 cells/mm^3^) 61.

Autologous HSCT, where indicated, is the treatment of choice for patients with HRL. It is important to ensure that there are still therapeutic options for HIV treatment before indicating HSCT for these patients. Most studies have shown that there is temporary HIV replication with an increased viral load and decreased CD4+ T-cell count shortly after the transplant procedure, but this period is rarely associated with HIV-related complications such as opportunistic infections. Patients with a detectable viral load or low CD4+ T-cell count should not be excluded from the HSCT procedure 60.

Data supporting the performance of allogeneic HSCT in HIV-infected patients are more limited than for autologous HSCT. Most of the current data are based on retrospective studies of a single institution including a small number of patients. The report of a case series from 1983 to 2010 observed significant improvement in the survival rates of HIV-infected patients undergoing allogeneic HSCT using HAART compared to those of patients without HAART 62. In a case series including five patients with hematologic malignancies who underwent allogeneic HSCT, the patients received tenofovir/emtricitabine in combination with either efavirenz (one patient) or raltegravir (four patients). There were no major complications, no need to switch HAART an no increased transplant-related mortality, and the patients maintained an undetectable HIV viral load 63.

One multicenter clinical trial included 17 HIV-infected patients with different hematologic disorders (acute myeloid leukemia, acute lymphocytic leukemia, myelodysplastic syndrome, and HRL) who underwent allogeneic HSCT. The 100-day related mortality was 0%, the graft-*versus*-host disease (GVHD) incidence was 41%, and the 1-year overall rate was 57% 64.

A large inpatient data set in the United States for HSCT from 1998 to 2012 showed no differences in inpatient mortality, healthcare-acquired infections and GVHD rates between HIV-positive (n=108 patients) and HIV-negative patients, and the study concluded that HSCT can be safely performed in HIV-positive patients 65. In allogeneic HSCT, however, HIV-positive patients had a significantly higher incidence of nontuberculous mycobacteria and cytomegalovirus infection than HIV-negative patients.

Therefore, as with autologous HSCT, allogeneic HSCT should also be considered as a standard therapeutic option for HIV-infected patients who meet the usual eligibility criteria for this procedure. It is suggested, however, to exclude patients with opportunistic infections who do not have therapeutic options for HIV treatment at the time of transplantation. Drug interaction may further hamper the management of these patients in the immediate posttransplantation period.

Several studies performing allogeneic HSCT in HIV-positive patients (based on the “Berlin Patient”) have been conducted with the intention of changing the natural history of HIV. The “Berlin Patient” underwent transplantation for treatment of acute myeloid leukemia with a donor with the delta32 mutation, which corresponds to the deletion of the CCR5 receptor from the cells. After 8 years of transplantation, the patient continues to have an undetectable HIV viral load in serum and in other tissues. However, another five patients who were transplanted with donors with the CCR5 deletion died 57,66,67.

### Pediatric Transplantation

Although the reported experience with transplantation in the HIV-positive pediatric population is limited to small case series reports, the concerns and challenges are similar to those in the adult population.

The main experience with SOT in HIV-positive children was published in 1990. Authors from the University of Pittsburgh reported 10 HIV-infected children among 25 SOT recipients in the pre-HAART period between 1981 and 1988. Five patients underwent transplantation after HIV infection (3 liver, 1 kidney, and 1 heart) 35. The median age of the children was 15 years (ranging from 6 months to 16 years), and 2 of them died posttransplantation. One died 3.5 years later from a systemic CMV infection attributed to AIDS, and the other died 9 months later from a preexisting central nervous system disorder that was not specified by the authors.

More recently, there was a report of a liver transplantation for a 9-year-old child with HIV who developed acute liver failure associated with efavirenz-based HAART; the patient was effectively treated with a combination of lamivudine, zidovudine and raltegravir 68.

Another group reported a successful case of kidney transplantation in an 8-year-old patient who developed nephropathy related to HIV infection that was treated with lamivudine, abacavir and lopinavir/ritonavir. The authors, however, highlighted the importance of carefully monitoring the blood tacrolimus level because of the drug interaction between the calcineurin inhibitors and protease inhibitors 69.

There is slightly more literature on HSCT than on SOT for HIV-positive children, although the majority of the pediatric HSCT cases have been reported together with adults who underwent autologous 70,71 or allogeneic transplantation 62,72.

There is a case report of successful high-dose chemotherapy, including rituximab, followed by autologous HSCT in a 13-year-old child with congenital HIV and refractory Burkitt lymphoma 73. At the time of hematopoietic transplantation, the patient was on his third antiretroviral regimen with lopinavir/ritonavir, lamivudine, tenofovir and stavudine, based on HIV genotypic drug resistance findings. Due to a temporary interruption of HAART during high-dose chemotherapy, there was a transient drop of the CD4+ count to 12 cells/mm^3^ and an increased viral load up to 585,000 copies/ml. After restarting antiretroviral treatment, the patients CD4+ count increased, and the viral load became undetectable. This patient remained in complete remission for a 26-month period.

Most of the reported cases have outlined uneventful posttransplantation evolution with no AIDS-related complications 60,62,70-73. Nevertheless, in contrast to the available data in adults, HSCT as part of a salvage strategy after high-dose chemo/radiotherapy in HIV-related NHL has not yet been established for children.

### Acceptability criteria for transplantation.

Current position for transplantation in HIV-positive candidates at the Hospital das Clínicas of the University of São Paulo:

### Kidney Transplant

Accept HIV-positive candidates: yes.

Specific criteria to accept the following:

- CD4+ T-lymphocyte count ≥200 cells/mm^3^ during at least 6 months before transplantation
  - Children: <1 year: CD4+ T-cell count ≥750 cells/mm^3^ or CD4+ T-cell percentage of total lymphocytes ≥26%; 1-5 years: CD4+ T-cell count ≥500 cells/mm^3^ or CD4+ T-cell percentage of total lymphocytes ≥22%; ≥6 years: the same criteria used for adults and adolescents.
- Undetectable HIV viral load in plasma using ultrasensitive techniques (<50 copies/mL) during at least 6 months before transplantation
- Use of the same HAART scheme during at least 6 months before transplantation
- Absence of active opportunistic infection and malignancy
- Absence of chronic wasting or severe malnutrition
- No evidence of advanced fibrosis or cirrhosis in patients with a history of HBV or HCV
- Appropriate follow-up with providers experienced in the management of HIV
  Specific criteria to reject:
- Patients with a previous history of the following opportunistic diseases under risk of further reactivation during the posttransplantation period in the absence of available target prophylaxis: progressive multifocal leukoencephalopathy, chronic intestinal cryptosporidiosis, primary central nervous system lymphoma, and visceral Kaposi’s sarcoma
- Patients with a previous history of antiretroviral resistance and no currently available antiretroviral drug options based on antiretroviral sensitivity testing can be considered a potential contraindication.

### Liver Transplant

Accept HIV-positive candidates: yes.

Specific criteria to accept the following:

- CD4+ T-lymphocyte count ≥100 cells/mm^3^ during at least 6 months before transplantation
  - If the patient presents intolerance to HAART due to severe terminal liver disease, transplant should be considered even with CD4+ T-cell ≤100 cells/mm^3^ and detectable viral load once there is a perspective of HIV control and immune reconstitution after reintroduction of HAART in the posttransplantation period.
  - Children: <6 years: CD4+ T-cell percentage of total lymphocytes ≥15%; ≥6 years: the same criteria as used for adults and adolescents.
- Undetectable HIV viral load in plasma using ultrasensitive techniques (<50 copies/mL) during at least 6 months before transplantation
- Use of the same HAART scheme during at least 6 months before transplantation
- Absence of active opportunistic infection and malignancy
- Absence of chronic wasting or severe malnutrition
- Appropriate follow-up with providers experienced in the management of HIV
  Specific criteria to reject:
- Patients with a previous history of the following opportunistic diseases at risk of further reactivation during the posttransplantation period in the absence of available target prophylaxis: progressive multifocal leukoencephalopathy, chronic intestinal cryptosporidiosis, primary central nervous system lymphoma, and visceral Kaposi’s sarcoma
- Patients with a previous history of antiretroviral resistance and no currently available antiretroviral drug options based on antiretroviral sensitivity testing can be considered a potential contraindication.

### Heart Transplant

Accept HIV-positive candidates: yes.

Specific criteria to accept the following:

- CD4+ T-lymphocyte count ≥200 cells/mm^3^ during at least 3 months before transplantation
  - Children: <1 year: CD4+ T-cell count ≥750 cells/mm^3^ or CD4+ T-cell percentage of total lymphocytes ≥26%; 1-5 years: CD4+ T-cell count ≥500 cells/mm^3^ or CD4+ T-cell percentage of total lymphocytes ≥22%; ≥6 years: the same criteria as used for adults and adolescents.
- Undetectable HIV viral load in plasma using ultrasensitive techniques (<50 copies/mL) during at least 3 months before transplantation
- Use of the same HAART scheme during at least 6 months before transplantation
- Absence of active opportunistic infection and malignancy
- Absence of chronic wasting or severe malnutrition
- No evidence of advanced fibrosis or cirrhosis in patients with a history of HBV or HCV
- Appropriate follow-up with providers experienced in the management of HIV

### Lung Transplant

Accept HIV-positive candidates: consider on a case-by-case basis.

There are limited data in the literature about the safety of lung transplantation in HIV patients. Despite the recent worldwide acceptance of HIV-positive patients for lung transplantation, the Lung Transplant Group of our hospital decided to individually evaluate HIV-positive candidates before listing for transplantation.

### Hematopoietic Stem Cell Transplant

Accept HIV-positive candidates: yes

Specific criteria to accept the following:

- CD4+ T-lymphocyte count ≥250 cells/mm^3^ during at least 6 months before transplantation
  - Children: <1 year: CD4+ T-cell count ≥750 cells/mm^3^ or CD4+ T-cell percentage of total lymphocytes ≥26%; 1-5 years: CD4+ T-cell count ≥500 cells/mm^3^ or CD4+ T-cell percentage of total lymphocytes ≥22%; ≥6 years: the same criteria as used for adults and adolescents.
- Undetectable HIV viral load in plasma using ultrasensitive techniques (<50 copies/mL) during at least 6 months before transplantation
  - Consider patients with a detectable HIV viral load to transplant in special situations
- Use of the same HAART scheme during at least 6 months before transplantation
- Absence of active opportunistic infection and malignancy

### Pediatric Transplant

Accept HIV-positive candidates: yes

Specific criteria to accept the following:

- Because there are insufficient data in the pediatric population, the recommendations should be according to the adult’s guidelines. However, we need to consider the absolute number of CD4+ T-cells in children based on their age (Table 1).

### Final consideration

Solid organ transplantation in HIV-positive patients has become an acceptable and safe procedure, with graft and patient survival rates equivalent to those of HIV-negative patients. The current acceptability criteria for transplantation developed for the Hospital das Clínicas of the University of Sao Paulo were based on the available reported data at the moment. These recommendations will be reviewed and may change based on new data that reinforce or refute the current evidence.

Transplantation for organs from HIV-infected donors to HIV-infected recipients is not allowed in Brazil, and this topic was not included in this manuscript. More data regarding the effectiveness and safety of this approach must be demonstrated to guide us in how we proceed with this issue.

## AUTHOR CONTRIBUTIONS

Pierrotti LC wrote the manuscript. All of the authors participated in the literature revision, discussion about the hospital position regarding transplantation criteria for HIV-positive patients and participated in the elaboration of the final manuscript. Odongo FC and Song AT reviewed English language. Sousa Marques HH and Abdala E approved the final version of the manuscript.

## Figures and Tables

**Table 1:** HIV infection stage based on age-specific CD4+ T-lymphocyte count or CD4+ T-lymphocyte percentage of total lymphocytes in children <13 years of age.

	Age on date of CD4+ T-lymphocyte test					
	<1 year		1-5 years		≥6 years	
Stage	Cells/mm^3^	%	Cells/mm^3^	%	Cells/mm^3^	%
1	>1,500	>34	≥1,000	≥30	**≥500**	>26
2	750-1,499	26-33	500-999	22-29	200-499	14-25
3	<750	<26	<500	<22	<200	<14

Adapted from: Centers for Disease Control and Prevention (CDC). Revised surveillance cases definition for HIV infection - United States, 2014. MMWR 2014;63(No. RR-3):1-10 (74)
